# Effects of Therapeutic Doses of Celecoxib on Several Physiological Parameters of Cultured Human Osteoblasts

**DOI:** 10.7150/ijms.37857

**Published:** 2019-09-20

**Authors:** Víctor J. Costela-Ruiz, Lucia Melguizo-Rodríguez, Rebeca Illescas-Montes, Javier Ramos-Torrecillas, Francisco J. Manzano-Moreno, Concepción Ruiz, Elvira De Luna- Bertos

**Affiliations:** 1 Biomedical Group (BIO277). Department of Nursing, Faculty of Health Sciences. University of Granada, Avda. Ilustración 60, 18016. Granada, Spain.; 2 Instituto Investigación Biosanitaria, ibs.Granada, C/ Doctor Azpitarte 4, 4ª planta, 18012. Granada, Spain.; 3 Biomedical Group (BIO277). Department of Stomatology, School of Dentistry, University of Granada, Colegio Máximo, Campus Universitario de Cartuja 18071. Granada, Spain.; 4 Institute of Neuroscience, University of Granada, Centro de Investigación Biomédica (CIBM). Parque de Tecnológico de la Salud (PTS) Avda. del Conocimiento S/N, 18016. Armilla, Granada, Spain.

**Keywords:** celecoxib, osteoblasts, COX-2 selective NSAIDs, osteoblast differentiation, bone.

## Abstract

Non-steroidal anti-inflammatory drugs (NSAIDs), including cyclooxygenase-2 (COX-2)-selective NSAIDs, are associated with adverse effects on bone tissue. These drugs are frequently the treatment of choice but are the least studied with respect to their repercussion on bone. The objective of this study was to determine the effects of celecoxib on cultured human osteoblasts. Human osteoblasts obtained by primary culture from bone samples were treated with celecoxib at doses of 0.75, 2, or 5μM for 24 h. The MTT technique was used to determine the effect on proliferation; flow cytometry to establish the effect on cell cycle, cell viability, and antigenic profile; and real-time polymerase chain reaction to measure the effect on gene expressions of the differentiation markers RUNX2, alkaline phosphatase (ALP), osteocalcin (OSC), and osterix (OSX). Therapeutic doses of celecoxib had no effect on osteoblast cell growth or antigen expression but had a negative impact on the gene expression of RUNX2 and OSC, although there was no significant change in the expression of ALP and OSX. Celecoxib at therapeutic doses has no apparent adverse effects on cultured human osteoblasts and only inhibits the expression of some differentiation markers. These characteristics may place this drug in a preferential position among NSAIDs used for analgesic and anti-inflammatory therapy during bone tissue repair.

## Introduction

Non-steroidal anti-inflammatory drugs (NSAIDs) include a large group of drugs that inhibit the cyclooxygenase enzyme (COX). These are divided between non-selective and selective inhibitors of COX-2 and are widely used in clinical practice to control pain and inflammation, especially in orthopedics and bone surgery 1,2.

Various *in vitro* studies have found that NSAIDs exert adverse effects on the physiology of osteoblasts, including: inhibition of their proliferative capacity and cell differentiation; alteration of their antigenic profile; and modulation of the gene expression of markers involved in cell growth and maturation and bone mineralization 3-9. In parallel, various *in vivo* studies have shown that these drugs affect bone tissue though a delay in bony callus formation in fractures 10,11 or a loss of bone mineral density 12, among others. However, research to date has mainly focused on non-selective NSAIDs.

Studies on the effect of COX-2 selective NSAIDs on bone tissue have largely been conducted in animal models and murine cell lines 13. There are currently no conclusive data on celecoxib, a potent selective NSAID mainly used in orthopedics and rheumatology. Some authors reported its adverse effects on bone formation in animal models 14, whereas others observed no celecoxib-related bone involvement 15.

The objective of this study was to determine the impact of celecoxib on cultured human osteoblasts by analyzing the effect of different celecoxib doses on the growth, antigenic profile, and gene expression of human osteoblasts obtained by primary culture from bone samples.

## Material and methods

### Primary Human Osteoblast

Human bone tissue was obtained from the Department of Stomatology of the University of Granada. All participants provided written informed consent to participate in this study, which was approved by the Ethical Committee of the University of Granada (Reg. No. 524/CEIH/2018). Primary osteoblasts were harvested from anonymous tissue in bone chips gathered during mandibular osteotomy or the surgical removal of lower wisdom teeth. We included a total of 3 donors in order to do all the assays for triplicate; 2 male and 1 female (30, 26 and 27 years old respectively). These cells were collected and cultured according to a standardized protocol 9, establishing three cell lines of primary culture human osteoblasts cultured. Cells were cultured until passage 4 to 6 before the treatment.

### Treatments

The osteoblastic cell lines were treated for 24 h with celecoxib (Sigma, St Louis, MO, USA) at doses of 0.75, 2, or 5 μM; untreated cells served as controls.

### Cell proliferation

Cell proliferation was determined by the MTT method as reported by Illescas-Montes et al. 16. Osteoblasts were seeded at 1 x 10^4^ cells/mL per well into a 96-well plate without fetal bovine serum (FBS) and cultured at 37 ºC for 24 h. The medium was then replaced with Dulbecco´s Modified Eagle medium (DMEM) containing celecoxib at a dose of 0.75, 2, or 5 μM. After 24 h, the medium was replaced with DMEM containing 0.5 mg/mL MTT (3-(4,5-Dimethylthiazol-2-yl)-2,5-diphenyltetrazolium bromide) supplied by Sigma and incubated for 4 h. Cellular reduction of the MTT tetrazolium ring resulted in the formation of a dark-purple water-insoluble deposit of formazan crystals. After incubation, the medium was aspirated and DMSO was added to dissolve the formazan crystals. Absorbance was measured at 570 nm with a spectrophotometer (SunriseTM, Tecan, Männedorf, Switzerland).

### Cell Cycle

Cultured human osteoblast cells treated with 0.75, 2, or 5 μM celecoxib or with no NSAID treatment (controls) were detached from the culture flask with a solution of 0.05 % trypsin (Sigma) and 0.02 % ethylene diamine tetraacetic acid (EDTA) (Sigma) and were then washed and suspended in phosphate buffered saline (PBS) and prepared for the cell cycle study by flow cytometry as previously reported 16.

### Apoptosis and necrosis analysis

Cultured human osteoblast cells treated with 0.75, 2, or 5 μM celecoxib for 24 h or with no NSAID treatment (controls) were detached from the culture flask, washed, suspended in 300 µL PBS, and then labeled with annexin V and propidium iodide (PI) (Immunostep S.L., Salamanca, Spain). We incubated 100 µL aliquots of the cell suspension with 5 µL annexin V and 5 µL PI for 30 min at 4 ºC in the dark. Cells were then washed, suspended in 1 mL PBS, and immediately analyzed in a flow cytometer with argon laser (Fasc Vantage Becton Dickinson, Palo Alto, CA) at a wavelength of 488 nm to determine the percentage of fluorescent cells. We calculated the percentage of annexin-positive (apoptotic) cells and PI-positive (necrotic) cells from counts of 2,000-3,000 cells.

### Antigenic phenotype

Antigenic phenotype was studied by flow cytometry at 24 h of culture after treatment with 0.75, 2, or 5 μM celecoxib. Untreated cells served as controls. Cells were detached from the cultured flask with 0.4 % (w/v) EDTA solution, washed, and suspended in PBS at 2×10^4^ cells/mL. Cells were labeled by direct staining with the monoclonal antibodies (MAbs) CD54, CD80, CD86, and HLA-DR (ICAM-1 monoclonal antibody [MEM-111], FITC; human CD80 [B7-1, BB1], FICT; human CD86 [B7-2, B70], FICT; and anti-human HLA-DR [Clas II], FICT; respectively, from Invitrogen, Thermo Fisher Scientific, Spain). Cells were subsequently analyzed by flow cytometry (FASC Canton II, SE Becton Dickinson, Palo Alto, CA) as previously described Melguizo-Rodriguez et al. 8.

### Gene expression determination by real-time polymerase chain reaction (RT-qPCR)

Gene expression analysis was performed as described Bustin et al. 17, Manzano-Moreno et al. 18 and Ragni et al. 19:

### RNA extraction and cDNA synthesis (reverse transcription)

After 24 h of culture with Celecoxib treatment (untreated cells served as controls), cells were detached from the culture flask using 0.05% trypsin-EDTA solution (Sigma) and individually harvested. mRNA was extracted using a silicate gel technique in the QiagenRNeasy extraction kit (Qiagen Inc., Hilden, Germany), which includes a DNAse digestion step. The amount of extracted mRNA was measured by UV spectrophotometry at 260 nm (Eppendorf AG, Hamburg, Germany), and contamination with proteins was determined according to the 260/280 ratio. An equal amount of RNA (1 μg of total RNA in 40 μl of total volume) was reverse-transcribed to cDNA and amplified by PCR using the iScript™ cDNA Synthesis Kit (Bio-Rad laboratories, Hercules, CA, USA), following the manufacturer`s instructions.

### Real-time polymerase chain reaction (RT-PCR)

Primers were designed using NCBI-nucleotide library and Primer3-design (Table 1) to detect mRNA of RUNX2, OSC, OSX, and ALP. All were matched to the mRNA sequences of target genes (NCBI Blast software).

Final results were normalized using ubiquitin C (UBC), peptidylprolyl isomerase A (PPIA), and ribosomal protein S13 (RPS13) as stable housekeeping genes.

Quantitative RT-PCR (q-RT-PCR) was conducted using the SsoFast™ EvaGreen® Supermix Kit (Bio-Rad laboratories) and following the manufacturer`s instructions. Samples were amplified in 96-well microplates in an IQ5-Cycler (Bio-Rad laboratories) at a specific annealing temperature for each gene, ranging from 60 to 65 ºC, and at an elongation temperature of 72 °C over 40 cycles. PCR reactions were carried out in a final volume of 20 μL, with 5 μL of cDNA sample and 2 μL of each primer. Ct values were plotted against log cDNA dilution to construct standard curves for each target gene. After each RT-PCR, a melting profile was created and agarose gel electrophoresis was conducted in each sample to rule out nonspecific PCR products and primer dimers. The comparative Ct method was employed for the relative quantification of gene expression. The mRNA concentration for each gene was expressed as ng of mRNA per average ng of housekeeping mRNAs. The cDNA from individual cell experiments was analyzed in triplicate RT-PCR studies.

### Statistical analysis

R software (version 2.9.2, Auckland, New Zealand) was used for data analyses. Mean values (±standard deviation) were calculated for each variable. A two-way repeated-measures analysis of variance (ANOVA) was performed to analyze the effects on proliferation, cell cycle, apoptosis/necrosis induction, and gene expression, considering treatment (celecoxib), time (24 h), and concentration (0.75, 2, or 5 μM). When significant interactions were identified, the Bonferroni correction was applied for planned pair-wise comparisons. P ≤ 0.05 was considered significant. Antigenic profiles were compared using the Student's t-test. P ≤ 0.05 was considered statistically significant in all tests. At least three experiments were performed in all assays and for each culture.

## Results

### Effect of celecoxib on osteoblast proliferation

**Figure 1:**
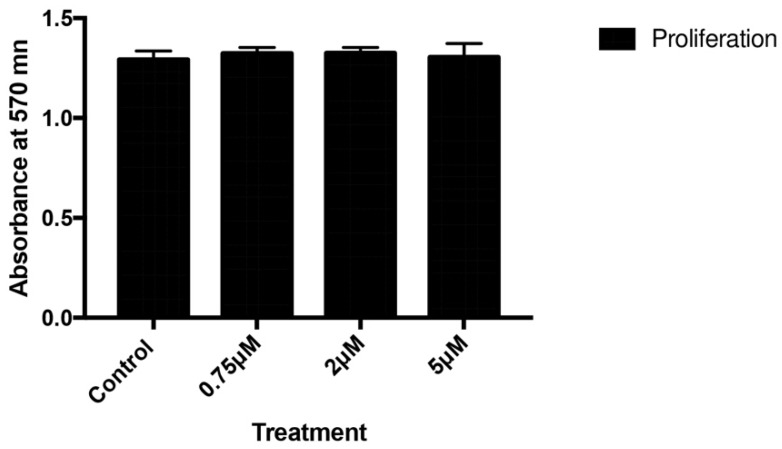
Effects of different celecoxib doses (0.75, 2, or 5 μM) on osteoblast proliferation after 24 h of treatment. Data are expressed as means +/± SD. Analysis of variance was used to compare data between each treatment and control culture.

Figure 1 depicts the effect of celecoxib on cell proliferation after 24 h of treatment with doses of 0.75, 2, or 5 µM. No significant changes in the proliferative capacity of osteoblasts were observed after treatment at any assayed dose of the drug.

### Effect of celecoxib on osteoblast cell cycle

**Figure 2:**
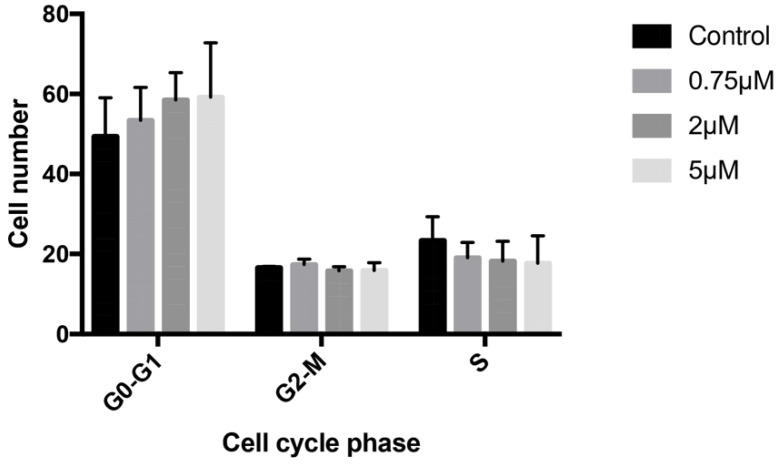
Effects of celecoxib on cell cycle determined by flow cytometry. Cultured cells were treated for 24 h with 0.75, 2, or 5 μM celecoxib. The control group was not treated. G0/G1, S, and G2/M represent the percentage of cells distributed among these phases after treatment, as determined by flow cytometry. Experiments were repeated at least three times.

Treatment with 0.75, 2, or 5 μM celecoxib for 24 h produced no significant changes in osteoblast cell cycle, finding similar cell percentages in G0/G1, G2/M, and S phases to those observed in the controls (Figure 2).

### Effect of celecoxib on osteoblast in apoptosis

No signs of cell apoptosis and/or necrosis were detected after osteoblast treatment with 0.75, 2, or 5 μM celecoxib for 24 h (Table 2).

### Effect of celecoxib on the expression of RUNX2, ALP, OSX, and OSC genes

**Figure 3:**
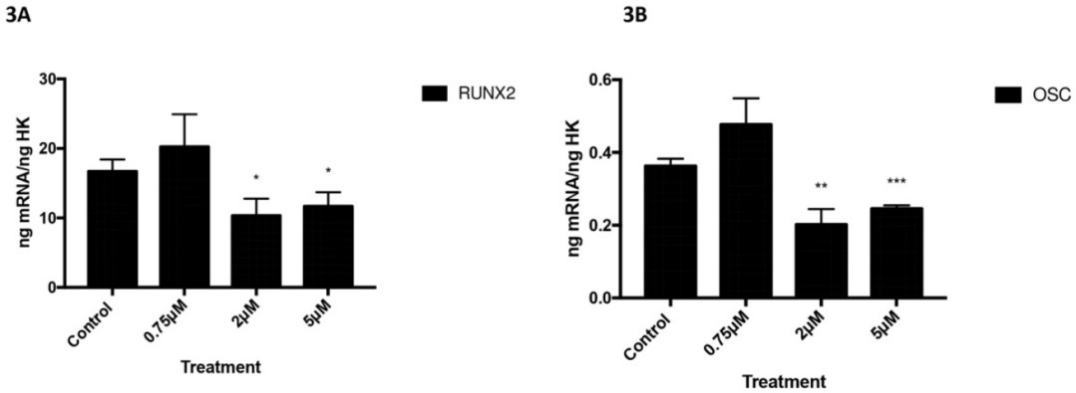
Expression of RUNX2 (3A) and OSC (3B) genes after 24 h treatment with 0.75, 2, or 5 µM. Data expressed as percentage expression with respect to control ± SD. (*** P ≤ 0.0001; ** P ≤ 0.01; * P ≤ 0.05).

**Figure 4:**
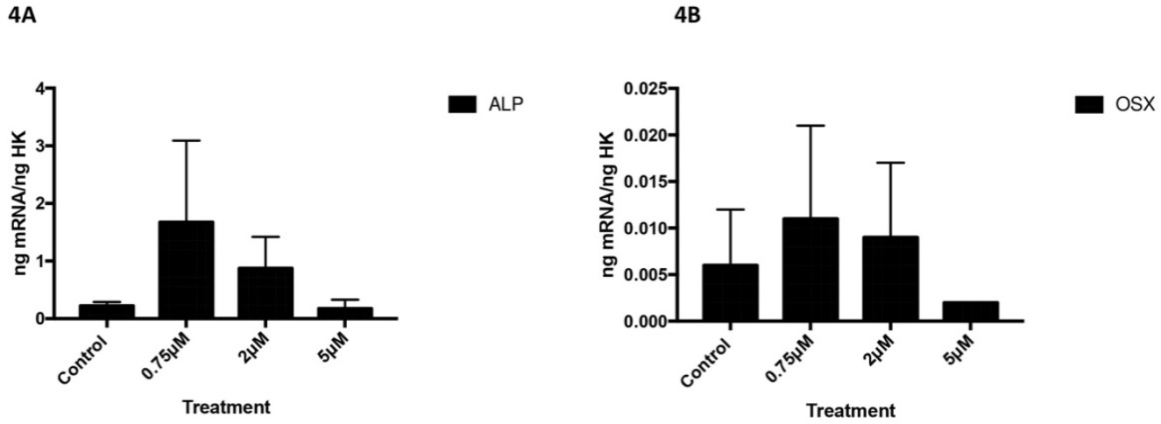
Expression of ALP (4A) and OSX (4B) genes after 24 h treatment with 0.75, 2, or 5 µM. Data expressed as percentage expression with respect to control ± SD. (*** P ≤ 0.0001; ** P ≤ 0.01; * P ≤ 0.05).

Figures 3 and 4 depict the q-RT-PCR results for the expression of osteoblast differentiation makers RUNX2, ALP, OSX, and OSC. After 24 h of treatment at a dose of 2 or 5 µM, osteoblast expression of RUNX2 (figure 3A) and OSC (figure 3B) was significantly lower *versus* controls (Figure 3).

However, no changes in the expression of these markers were observed at the lowest dose (0.75 µM). ALP (Figure 4A) and OSX (Figure 4B) expression did not change after treatment with celecoxib at any assayed dose (Figure 4).

### Effect of celecoxib on phenotypic profile

**Figure 5:**
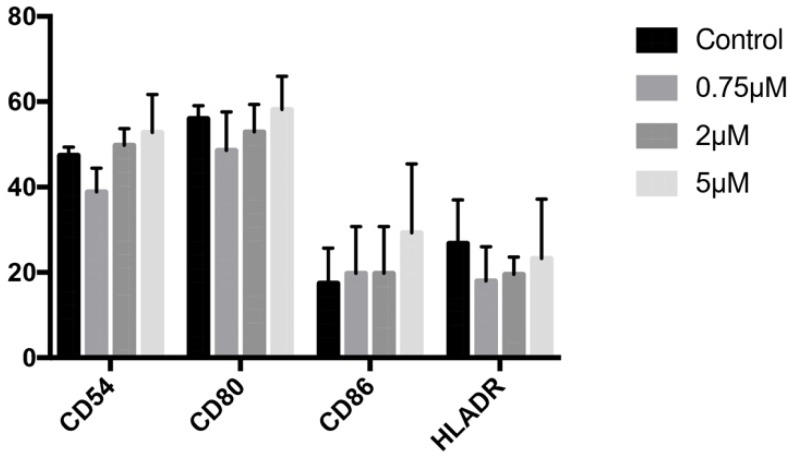
Expression of CD54, CD80, CD86, and HLA-DR genes by cells treated for 24 h with celecoxib (0.75, 2, or 5 μM). Data are expressed as percentage expression with respect to control ±SD.

Expression of CD54, CD80, CD86, or HLA-DR genes did not significantly differ between cells treated for 24 h with 0.75, 2, or 5 µM celecoxib and untreated control cells (Figure 5).

## Discussion

Treatment of primary culture human osteoblasts with therapeutic doses of celecoxib, a COX-2 selective NSAID, had no significant effects on their antigenic profile, growth, proliferation, or cell cycle and produced no signs of cell death. However, this treatment reduced the gene expression of RUNX2 and OSC, two biological markers with an important role in osteoblast differentiation, whereas no changes were observed in the expression of ALP or OSX. These results suggest that celecoxib, unlike other NSAIDs 7, acts on the expression of some of the genes involved in cell differentiation but does not affect other cell parameters such as growth or antigen expression.

The finding that celecoxib at 0.75, 2, or 5 µM (within the therapeutic range) has no effect on osteoblast growth is in line with previously published results. For instance, proapoptotic effects were observed after the treatment of MG63 human osteoblast cells with celecoxib at a dose of 50 µM but not at a dose of 2 or 10 µM 20, and no changes in the proliferation or viability of the murine pre-osteoblastic cell line MC3T3-E1 were observed after treatment with celecoxib at a dose of 20 µM 21. However, celecoxib at doses of 0.1 or 1 µM was found to inhibit cell proliferation and arrest the cell cycle in G0/G1 phase in cultured human osteoblasts 4 and in mesenchymal cells derived from mouse bone marrow 22. This last effect has been reported for other non-selective NSAIDs, in which the drug dose plays an important role 5,6,23.

The above effects of celecoxib on osteoblasts *in vitro* are closely related to observations in experimental animals. Thus, Kasukawa et al. 15 treated ovariectomized mice at a dose of 4 mg/kg for four weeks and found no change in bone formation but observed an effect on C- telopeptide, a marker related to resorption, a function carried out at bone level by osteoclasts. Liu et al. 24 treated *Sprague Dawley* rats with daily doses of 50 mg/kg celecoxib for two weeks and also observed no significant effect on bone formation.

Celecoxib does not appear to compromise bone healing, given that bone fusion was not altered by its administration at 10 mg/kg/day for eight weeks to rabbits after posterolateral intertransverse arthrodesis 25. In another study, Brown et al. 26 treated male rats with 3 mg/kg/day celecoxib or 1 mg/kg/day indomethacin for four or eight weeks and found no difference in healing *versus* controls for the celecoxib-treated rats but a delay in healing for the indomethacin-treated animals. Osteoblasts are directly involved in bone fracture healing, being the cells responsible for bone formation and repair during this process 27,28.

Osteoblasts have a characteristic antigenic profile, including antigens whose expression is modulated by the degree of cell maturation and/or the presence of certain biomolecules such as proinflammatory cytokines 29,30. Various NSAIDs have been found to modulate the expression of CD54, CD80, and HLA-DR 5,23,31. However, celecoxib treatment did not change the expression of these markers at any dose assayed in the present study.

Although no effect on osteoblast growth or antigenic profile was observed, celecoxib doses of 2 and 5 µM inhibited the gene expression of differentiation markers RUNX2 and OSC but not ALP or OSX. Matsuyama et al. 21 reported that celecoxib doses between 0.02 and 20 µM inhibit osteoblast differentiation in the MC3T3-E1 line but do not alter cell viability, indicating that negative effects on bone tissue at doses above 0.02 μM may be attributable to the inhibition of osteoblast differentiation. Nagano et al. 32 found that 50 µM celecoxib can inhibit the expression of RUNX2 mRNA in the same cell line (MC3T3-E1).

OSC is a peptide hormone secreted by osteoblasts during bone formation at a late stage of osteoblast differentiation, and it participates in bone mineralization and osteoclast activity regulation 33. It is transcribed by the RUNX2/CBFA1 complex 34. This raises the question whether the decrease in OSC expression is directly caused by the celecoxib treatment or is indirectly produced by its inhibitory effect on the expression of RUNX2. Celecoxib was found to negatively affect bone formation in mice, observing a reduced expression of OSC mRNA in bone marrow samples from those administered with 30 mg/kg celecoxib twice daily for five days 14.

Selective and non-selective NSAIDs are used in the treatment of heterotopic ossification, which can appear after surgery, especially arthroplasty. This ossification of soft tissue results from an abnormal differentiation to bone cells of mesenchymal stem cells. NSAIDs are used to inhibit the bone differentiation of progenitors and prevent this surgical complication 35-38. These data may be related to our finding of a celecoxib-induced decrease in the gene expression of markers such as RUNX2 and OSC. However, celecoxib treatment was found to reduce inflammation in rats with collagen-induced arthritis, and high doses of this drug prevented bone loss adjacent to inflamed joints and significantly decreased bone resorption but had no impact on bone formation 39. The authors concluded that celecoxib had a preventive effect on both growth plate destruction and bone loss adjacent to inflamed joints in this arthritis model.

In summary, our study shows that therapeutic doses of celecoxib, ranging from 0.75 to 5 μM, have no adverse effects on osteoblast growth or antigen expression but reduce the gene expression of RUNX2 and OSC. It is not possible to establish whether this reduction affects physiological bone development given the absence of changes in the expression of ALP or OSX, also involved in differentiation. Further studies are required to determine whether celecoxib interferes with the maturation of osteoblasts and therefore with their function at bone tissue level. Nevertheless, our results indicate that this NSAID is a good option for analgesic and anti-inflammatory therapy during bone tissue repair.

## Figures and Tables

**Table 1:** Primer sequences for the amplification of cDNA by real-time PCR.

Gene	Sense primer	Antisense primer	Amplicon (bp)
RUNX2	50 -TGGTTAATCTCCGCAGGTCAC-30	50 -ACTGTGCTGAAGAGGCTGTTTG-30	143
OSX	50 -TGCCTAGAAGCCCTGAGAAA-30	50 -TTTAACTTGGGGCCTTGAGA-30	205
ALP	50 -CCAACGTGGCTAAGAATGTCATC-30	50 -TGGGCATTGGTGTTGTACGTC-30	175
OSC	50 -CCATGAGAGCCCTCACACTCC-30	50 -GGTCAGCCAACTCGTCACAGTC-30	258

**Table 2:** Results of apoptosis assay in cultured cells treated for 24 h with celecoxib doses of 0.75, 2, or 5 μM.

	Necrotic cells			Viable cells			Apoptotic cells		
	Mean	P Value	SD	Mean	P Value	SD	Mean	P Value	SD
Control	0,633	-	0,321	91,233	-	10,404	8,133	-	10,082
0,75	0,466	0,519	0,251	91,666	0,952	5,396	7,866	0,970	5,492
2	0,366	0,230	0,057	83,566	0,295	3,655	16,066	0,269	3,611
